# Genome-wide association study and genomic selection for tolerance of soybean biomass to soybean cyst nematode infestation

**DOI:** 10.1371/journal.pone.0235089

**Published:** 2020-07-16

**Authors:** Waltram Second Ravelombola, Jun Qin, Ainong Shi, Liana Nice, Yong Bao, Aaron Lorenz, James H. Orf, Nevin D. Young, Senyu Chen

**Affiliations:** 1 Department of Horticulture, PTSC316, University of Arkansas, Fayetteville, AR, United States of America; 2 Hebei Academy of Agricultural and Forestry Sciences, Shijiazhuang, Hebei, China; 3 Southern Research & Outreach Center, University of Minnesota, Waseca, MN, United States of America; 4 Department of Agronomy and Plant Genetics, University of Minnesota, St. Paul, MN, United States of America; 5 Department of Plant Pathology, University of Minnesota, St. Paul, MN, United States of America; University of Guelph, CANADA

## Abstract

Soybean cyst nematode (SCN), *Heterodera glycines* Ichinohe, is one of the most devastating pathogens affecting soybean production in the U.S. and worldwide. The use of SCN-resistant soybean cultivars is one of the most affordable strategies to cope with SCN infestation. Because of the limited sources of SCN resistance and changes in SCN virulence phenotypes, host resistance in current cultivars has increasingly been overcome by the pathogen. Host tolerance has been recognized as an additional tool to manage the SCN. The objectives of this study were to conduct a genome-wide association study (GWAS), to identify single nucleotide polymorphism (SNP) markers, and to perform a genomic selection (GS) study for SCN tolerance in soybean based on reduction in biomass. A total of 234 soybean genotypes (lines) were evaluated for their tolerance to SCN in greenhouse using four replicates. The tolerance index (TI = 100 × Biomass of a line in SCN infested / Biomass of the line without SCN) was used as phenotypic data of SCN tolerance. GWAS was conducted using a total of 3,782 high quality SNPs. GS was performed based upon the whole set of SNPs and the GWAS-derived SNPs, respectively. Results showed that (1) a large variation in soybean TI to SCN infection among the soybean genotypes was identified; (2) a total of 35, 21, and 6 SNPs were found to be associated with SCN tolerance using the models SMR, GLM (PCA), and MLM (PCA+K) with 6 SNPs overlapping between models; (3) GS accuracy was SNP set-, model-, and training population size-dependent; and (4) genes around *Glyma*.*06G134900*, *Glyma*.*15G097500*.*1*, *Glyma*.*15G100900*.*3*, *Glyma*.*15G105400*, *Glyma*.*15G107200*, and *Glyma*.*19G121200*.*1* (Table 4). *Glyma*.*06G134900*, *Glyma*.*15G097500*.*1*, *Glyma*.*15G100900*.*3*, *Glyma*.*15G105400*, and *Glyma*.*19G121200*.*1* are best candidates. To the best of our knowledge, this is the first report highlighting SNP markers associated with tolerance index based on biomass reduction under SCN infestation in soybean. This research opens a new approach to use SCN tolerance in soybean breeding and the SNP markers will provide a tool for breeders to select for SCN tolerance.

## Introduction

Soybean [*Glycine max* (L.) Merr.] is a widely grown legume with high oil and protein contents. The wild type *Glycine soja* Sieb. & Zucc. was used for soybean domestication [1]. Soybean is one of the most economically important cultivated legumes worldwide. The value of biofuel made from soybean was reported to exceed $35 billion in the United States (www.soystats.com). Increase in need for soybean production has been significant [2]. This requires the use of high-yielding soybean cultivars and the expansion of croplands for soybean cultivation. However, soybean production has been constrained by various factors. Soybean cyst nematode, *Heterodera glycines* Ichinohe, has been one of the most devastating biotic stresses affecting soybean production worldwide. Costs associated with soybean production loss due to SCN have exceeded $1.5 billion in the U.S. alone [3].

The soybean cyst nematode (SCN) is an obligate parasite and has been found in the most soybean-producing areas in the U.S. [4]. SCN feeds on soybean roots and uses soybean plants as a carbon source. This will cause a decrease in soybean biomass and suppress soybean yield [5]. SCN is difficult to control upon establishment in fields. One of the most effective ways to manage SCN is the use of SCN-resistant soybean cultivars and a non-host plant during crop rotation [6]. Therefore, developing new SCN-resistant soybean cultivars through breeding is of great importance.

Breeding for new SCN-resistant soybean cultivars requires a better understanding of the genetic mechanism conferring SCN resistance. A total of 216 QTLs in soybean have been identified to confer resistance to SCN (www.soybase.org). Of which, two loci were extensively investigated. These two loci consisted of *rhg1* and *Rhg4*, which were mapped on chromosomes 18 and 8, respectively [7]. The soybean cultivar ‘Forest’ harbored both SCN-resistant QTLs, and *Rhg4* is dominant [8]. This resistance comes from the Peking accession. The second type of resistance only requires *rhg1* and this type of resistance comes from PI 88788 [9]. The *Rhg4* locus contained a gene encoding for a serine hydroxymethyltransferase [10], whereas the genes within the *rhg1* locus encoded for an amino acid transporter, an α-soluble N-ethylmaleimide-sensitive factor attachment protein (α-SNAP), and a wound-inducible domain protein (WI12) [11].

The development of cultivars enhanced with disease resistance has been time-efficiently achieved thanks to the use of molecular markers via marker-assisted selection (MAS) [12]. With the recent development of high-throughput sequencing technology, tools such as genome-wide association study (GWAS) and genomic selection (GS) have been proven to be powerful for investigating the genetic architecture of complex traits [13]. Previous studies demonstrated that GWAS can be used to efficiently identify single nucleotide polymorphism (SNP) markers associated with SCN resistance in soybean. A total of 7 SNP markers were reported to be associated with resistance to SCN HG type 0 using GWAS [14]. In addition, two new candidate genes, *FGAM1* and *Glyma18g46201*, were identified to be associated with SCN resistance [14]. In another GWAS, a total of 440 soybean accessions were phenotyped for resistance to SCN HG type 0 and HG type 1.2.3.5.7, and 19 SNP markers were shown to be associated with SCN resistance [15]. In addition, a GWAS conducted on a panel consisting of 553 soybean accessions identified a total of 8 new loci contributing to resistance to SCN [16]. Predictive breeding using genomic selection has brought considerable attention over the past few years. Genomic selection (GS) was reported to be more efficient over marker-assisted selection (MAS) for SCN resistance in soybean [14]. The earliest GS study for SCN resistance suggested an accuracy in the range of 0.59 to 0.67 for predicting SCN resistance in soybean [14].

The current commercial U.S. soybean cultivars have a narrow genetic background [1]. Due to this narrow genetic background, the current soybean germplasm would be vulnerable to nematode infestation [7, 17]. This could be addressed by diversifying the source of resistance to nematodes and by investigating potential new loci conferring SCN resistance. Evaluating tolerance index based on biomass reduction under SCN infestation and characterizing loci affecting such trait could lead to a new approach for breeding SCN tolerance in soybean. Therefore, the objectives of this study were (i) to conduct GWAS for tolerance of soybean biomass to SCN infection, (ii) to identify SNP markers associated with SCN tolerance based on decrease in plant biomass, and (iii) to perform GS for soybean tolerance to SCN. The trait of SCN tolerance is different from the trait of SCN resistance in that a tolerant soybean can support good SCN reproduction but suffer little damage from the SCN infection, while SCN-resistant soybean does not support SCN reproduction.

## Materials and methods

### Plant materials and phenotyping

The association panel investigated in this study consisted of 234 soybean accessions (S1 Table) from the panel of 288 lines used for a previous GWAS of SCN resistance [14]. A large number of these accessions were selection from the University of Minnesota soybean breeding program and 9 were Plant Introductions (PIs). Ten lines of the panel were resistant and 6 lines were moderately resistant to SCN HG Type 0 (race 3) with the resistance from PI 88788 (*rhg1*). ‘MN0095’ was used as a susceptible check and a few lines derived from PI 88788 harboring SCN-resistant genes were used as resistant checks [14, 18].

SCN phenotyping was carried out in the greenhouse of the University of Minnesota St. Paul campus. Soil without SCN infestation collected from a soybean field was mixed with sand at a 2:1 ratio, and 1.5 kg of the soil-sand mixture was placed in 1-galon plastic bags. The natural field soil rather than the sterilized soil was used because the data from the natural field soil would be better for extrapolating the results to the field setting. Particularly, we considered the importance of rhizobium for the soybean growth, and the natural field soil can support sufficient rhizobium development. The soil from each bag was used in one 16-cm-diam clay pot. Soybean cyst nematode HG Type 1.2.3.5.6.7 (race 4), which can reproduce well on the lines containing resistance genes from PI 88788, was used. The SCN eggs at a density of 10,000 eggs/100 cm^3^ of soil and diluted into 10 ml water were added into the soil in each SCN pot. Ten soybean seeds were placed on the surface each pot and the seeds were covered with the remaining soil. Four replicated pots were included for each soybean accession in both SCN and no-SCN treatments. The two pots (SCN and no-SCN) of the same soybean line were placed together to minimize the environmental difference between the SCN and no-SCN treatments within a genotype. Due to the large number of lines and limitation of the space of the greenhouse, this experiment was conducted at four different times with approximately 60 lines per time in the same greenhouse. Although lines of each replicate were arranged in a randomized block (S1 and S2 Figs), the experiment was considered complete randomized design because the lines were evaluated in four groups at four different times. The distances between each two pots were about 10 cm (S2 Fig).

After 5 days, the plants were thinned to provide five plants per pot. At 65 days after planting, the average total dry shoot biomass of the five plants in each pot under non-SCN infestation or with SCN infestation was measured. Tolerance index for biomass was computed using the following formula [19].

Tolerance Index (TI) = 100 × (Biomass under SCN infestation/Biomass without SCN infestation)

TI values were adjusted to that of ‘MN0095’, used as susceptible controls, in order to minimize environmental effects within and between runs. In each run, there were two sets of ‘MN0095’ pots with total of 8 pairs of pots inoculated with SCN or not inoculated.

TI_adjusted = TI × (Average TI for ‘MN0095’ between runs/Average TI for ‘MN0095’ within each run) with average TI for ‘MN0095’ between runs/average TI for ‘MN0095’ within each run being the adjustment coefficient. There were a total of 936 TI data points (234 soybean lines x 4 replicates). ANOVA on TI_adjusted values was performed using PROC MIXED of SAS v. 9.4 (SAS Institute Inc., Cary, NC, USA). Mean separation was conducted using a protected LSD procedure at a significance level α = 0.05. The statistical model for ANOVA analysis was described as following.

$$Y_{ij}=R_{i}+G_{j}+\varepsilon_{ij}\;with\;i=1,2,3\;and\;j=1,\ldots .,234$$

where Y_ij_ denoted the observation on the j^th^ genotype from the i^th^ run, R_i_ represented the effect of the i^th^ replication (random effect), G_j_ was the effect of the j^th^ soybean accession (fixed effect), and ε_ij_ was the random error associated with the ij^th^ observation. Broad sense heritability was calculated using *H* ^*2*^ = 100 × (σ^2^_g_/σ^2^_p_) = 100 × σ^2^_g_/[σ^2^_g_ + (σ^2^_e_/r)] [20] where σ^2^_g_ was the genotypic variance (σ^2^_g_ = MSGenotype-MSError), σ^2^_p_ denoted the total phenotypic variance, σ^2^_e_ was the variance associated with the random error, and r represented the number of replications. Graph showing the data distribution was drawn using JMP® oGenomics 9 (SAS Institute, Cary, NC).

### Genotyping and quality control

The soybean panel was genotyped using the Soy6K SNP Infinium Chips (https://www.soybase.org/snps/download.php). DNA was extracted from young leaves of each accession using DNeasy 96 Plant Kit (QIAGEN, Valencia, CA). A total of 4,251 SNPs were obtained. Of the 4,251 SNPs, a total of 3,782 SNPs were maintained after SNP filtering (missing data<15%, heterozygosity<20%, minor allele frequency>5%). Those high-quality SNPs were used for further analysis.

### Genome-wide association study (GWAS) and candidate gene discovery

Genome-wide association study was conducted using TASSEL 5 [21]. A total of 3 GWAS statistical models were used. These models consisted of single marker regression (SMR), generalized linear model using principal component (PCA) as additional covariate (GLM_(PCA)), and mixed linear model using principal component (PCA) and Kinship (K) as covariates (MLM_(PCA+K)). The LOD threshold for declaring a significant SNP was 3 [22]. The 50-kb genomic region containing the significant SNP was used for the candidate gene(s) search. Functional annotation related to the candidate gene(s) was investigated using Soybase (www.soybase.org). Candidate genes related to plant defense mechanism were more considered.

### SNP selection accuracy and efficiency

SNP selection accuracy and efficiency were computed using the formulas established by Shi et al. [23] as shown below.

- *Selection accuracy* = 100×[(Number of genotypes having high tolerance index with the favorable SNP allele)/ (Number of genotypes having high tolerance index with the favorable SNP allele + Number of genotypes having low tolerance index with the favorable SNP allele)].
- *Selection efficiency* = 100×[(Number of genotypes having high tolerance index with the favorable SNP allele)/(Total number of genotypes having the favorable SNP allele)].

The top 78 SCN-resistant soybean genotypes (one-third of the whole panel) were the genotypes having high tolerance index, whereas the 78 least performing genotypes (one-third of the whole panel) had low tolerance index.

### Genomic estimated breeding values (GEBVs) and genomic selection accuracy assessment

Genomic estimated breeding values were computed under 5 different genomic selection models: ridge regression best linear unbiased predictor (rrBLUP) [24], genomic best linear unbiased predictor (gBLUP) [25], Bayesian least absolute shrinkage and selection operator (Bayesian LASSO) [26], random forest [27], and support vector machines (SVMs) [28]. The packages ‘rrBLUP’ [29], GAPIT [30], ‘BGLR’ [31], ‘randomForest’ [32], and ‘kernlab’ [33] were used and run in R to perform the genomic selection models rrBLUP, gBLUP, Bayesian LASSO, random forest, and SVMs, respectively. The posterior distribution of the parameter in the Bayesian LASSO model was Double Exponential and the prior distributions were Uniform and Inverse Chi-Square for the hypoparameters λ and σ^2^_e_. Markov Chain Monte Carlo (MCMC) iteration and the burin were set to 5,000 and 1,000, respectively, when running the Bayesian LASSO model [34]. Random forest was achieved using a total of 500 trees and 4 branches for each tree as previously described [14]. The SVMs model was done using a Gaussian kernel function [33].

In order to assess the effect of population training size on genomic selection accuracy of tolerance index based on biomass reduction under SCN infestation, cross-validation was conducted at different levels. In this study, we have performed a 2-fold, 3-fold, 4-fold, 5-fold, 6-fold, and 7-fold cross-validation corresponding to a training population size of 117, 156, 176, 187, 195, and 201 individuals, respectively. A total of 100 replications were conducted at each level of cross-validation. Genomic selection was conducted using all filtered SNPs and the selected SNPs from GWAS under the single marker model (SMR_SNPs), the generalized linear model (GLM_PCA_SNPs), and the mixed linear model (MLM_PCA_K_SNPs), respectively. In order to better fit the genomic selection model when the GWAS-derived SNPs were used, the number of covariates (SNPs) was increased by choosing the SNPs with LOD greater than 2 instead of 3. Fewer SNPs incorporated into the models would result in poorly fitted genomic selection models. Genomic selection accuracy was estimated by evaluating the Pearson’s correlation coefficient between the GEBVs and the observed phenotypes in the testing set [35].

## Results

### Phenotyping

**Fig 1:**
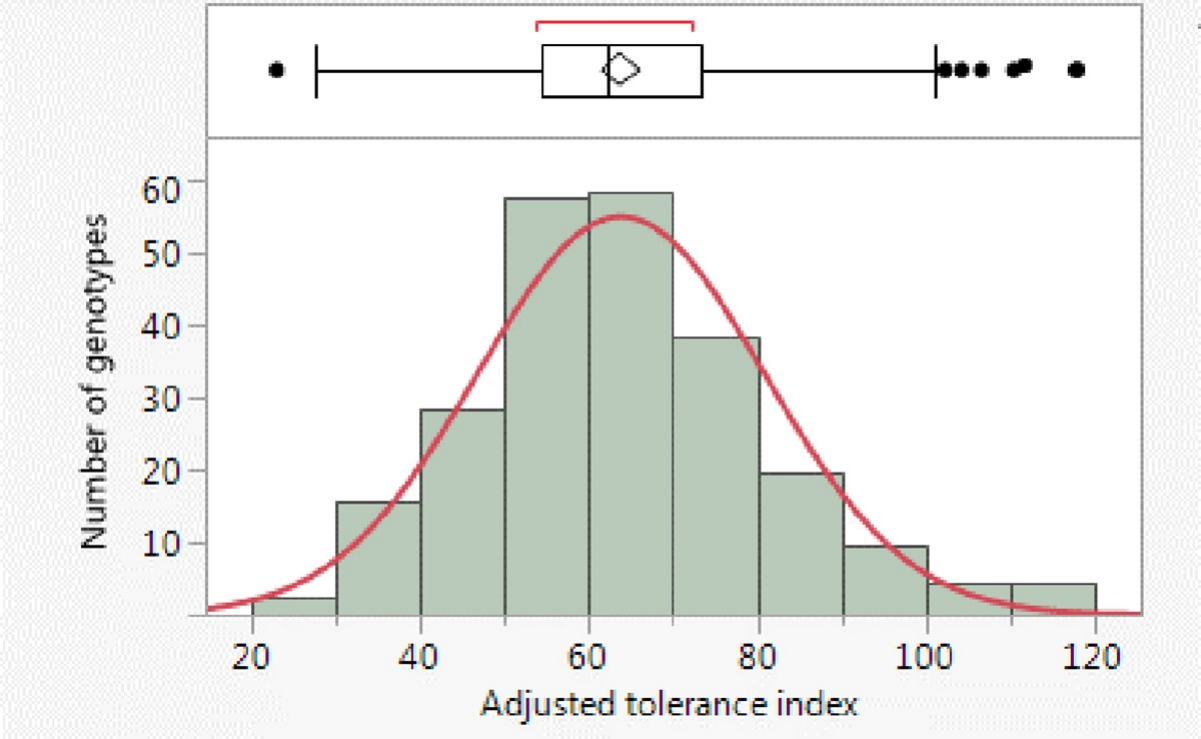
Distribution of adjusted tolerance index among the 234 soybean accessions.

Adjusted tolerance index for biomass reduction for the 234 soybean accessions was approximately normally distributed (Fig 1). The tolerance index was used to measure the level of tolerance of soybean to the SCN infection. The higher the index was, the more tolerant to SCN infection the genotype was.

Adjusted tolerance index varied from 22.87 to 118.16, with an average of 63.80 and a standard deviation of 17.03 (S1 Table). The analysis of variance (ANOVA) indicated that adjusted tolerance index was statistically significantly different among the soybean accessions (F value = 3.35, p-value<0.0001) (Table 1). Broad sense heritability was estimated using the variance components from ANOVA (Table 1). Estimate of broad sense heritability for adjusted tolerance index was high (89.3).

**Table 1:** ANOVA table for tolerance index based on biomass reduction under SCN infestation among 234 soybean genotypes.

Source	DF	Sum of Squares	Mean Square	Expected Mean Square	F Value	Pr > F
**Accession**	233	265549	1139.70	Var(Residual) + Q(Accession)	3.35	< .0001
**Rep**	3	2224.29	741.43	Var(Residual) + Q(Rep)	2.12	0.096
**Residual**	669	246400	368.31	Var(Residual)	.	

The lowest adjusted tolerance index was recorded for MN0082SP (22.87), PI445799 (27.49), PI437267 (30.10), PRIDEB216 (30.50), M95228092 (31.88), M95274129 (32.16), L237 (32.43), M95274114 (33.63), M95227016 (34.50), and M94278001 (35.11) (S1 Table), indicating that SCN infestation resulted in a significant reduction in biomass for those genotypes, thus intolerant to SCN. The genotypes with the highest adjusted tolerance index were GRANDE (98.68), M97251029 (99.70), ALTONA (101.29), MN1804CN (102.37), M97305077 (104.35), M97304052 (106.70), M97205096 (110.55), M98332108 (117.86), MN1806SP (117.88), and ALPHA (118.16) (S1 Table), indicating that SCN infestation did not significantly reduce biomass for those accessions, hence they were SCN-tolerant.

### SNP profiling

After SNP filtering, a total of 3,782 high quality SNPs were used for GWAS. Average number of SNPs per chromosome was 189, with chromosome 18 having the highest number of SNPs (256) and chromosome 11 harboring the lowest number of SNPs (127) (Table 2). Average distance between SNP was 255 kb. SNP density was the highest on chromosome 13 with an inter-SNP distance of 181 kb (Table 2). SNPs were most scattered on chromosome 1 with an average distance of 374 kb between SNPs (Table 2). Kinship analysis revealed that the population was structured into 3 subgroups (S3 Fig), of which, two subgroups had similar size.

**Table 2:** SNP profiling used for GWAS for tolerance to SCN infection based on biomass reduction.

Chromosome	SNP number	Average distance (kb) between SNPs
1	150	374
2	239	216
3	180	267
4	178	277
5	188	222
6	191	264
7	198	226
8	201	229
9	177	265
10	185	277
11	127	302
12	148	271
13	245	181
14	185	269
15	225	224
16	160	235
17	171	245
18	256	244
19	184	276
20	194	241

### Genome-wide association study (GWAS)

#### Single marker regression (SMR)

**Fig 2:**
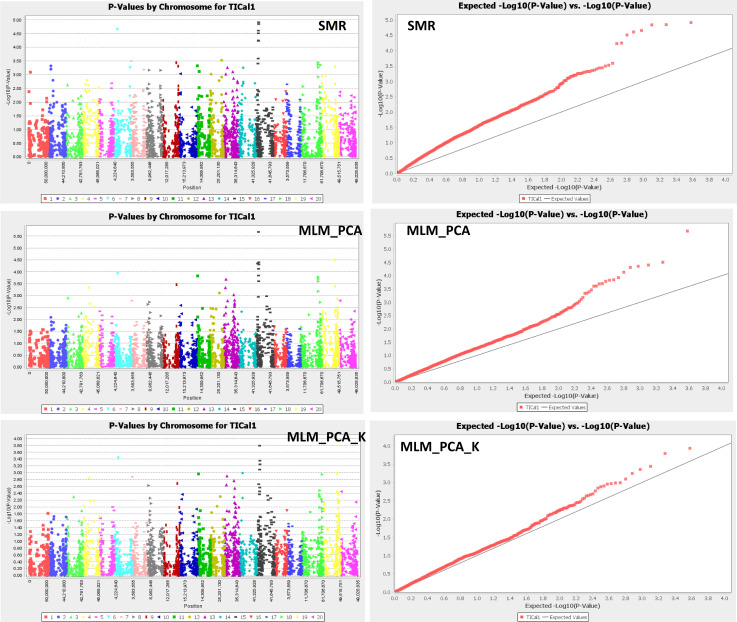
Manhattan plots and QQ-plots for tolerance indexes based on biomass reduction under SCN infestation. The x-axis of each Manhattan plot represented the chromosome number, whereas the y-axis denoted the LOD (-log10(p-value)). Color coding on the Manhattan plot is chromosome-wise. The x-axis of each QQ-plot represented the expected -log10(p-value), whereas the y-axis displayed the observed -log10(p-value). **A**: Manhattan plot and QQ-plot resulted from the single marker regression model (SMR). **B**: Manhattan plot and QQ-plot obtained using the generalized linear model (GLM(PCA)). **C**: Manhattan plot and QQ-plot generated by the mixed liner model (MLM(PCA+K)).

The single marker regression model indicated a total of 35 significant SNPs (LOD>3) associated with tolerance index based on biomass reduction under SCN infestation (Table 3) (Fig 2A). These significant SNPs were scattered across the soybean genome. The top 5 SNPs with the highest LOD were Gm15_8263547_G_T (LOD = 4.92, MAF = 13.60%), Gm15_8412363_G_A (LOD = 4.85, MAF = 12.89%), Gm19_37932358_C_T (LOD = 4.84, MAF = 48.69%), Gm06_11098210_C_T (LOD = 4.66, MAF = 16.67%), and Gm15_7864348_G_T (LOD = 4.61, MAF = 11.21%), which were found on chromosomes 15, 15, 19, 6, and 15, respectively (Table 3) (Fig 2A). These SNPs had relatively low individual R-square values ranging from 9.09% to 10.01% (Table 3), suggesting a possibility of QTL(s) with small effects affecting tolerance index based on biomass reduction under SCN infestation. However, if the marker effects of the 5 top SNPs were pooled under an additive model assumption, the effects could account for up to 47% of the variation in tolerance index based on biomass reduction.

**Table 3:** Significant SNPs (LOD>3.00) associated with tolerance to biomass reduction under SCN infestation using a Single Marker Regression (SMR), Generalized Linear Model_PCA (GLM_(PCA)), and Mixed Liner Model_PCA_K(MLM_(PCA+K)) models.

Statistical_model	SNP_marker	Chromosome	Position_(bp)	LOD (-log_10_(p-value))	R_square (%)	Minor_allele_frequency(%)
SMR	Gm01_4726775_C_A	1	4726775	3.09	6.47	23.61
	Gm02_8081081_T_C	2	8081081	3.20	6.51	38.29
	Gm02_8273157_G_A	2	8273157	3.32	6.77	35.29
	Gm02_8341731_C_T	2	8341731	3.20	6.72	36.74
	Gm06_11098210_C_T	6	11098210	4.66	9.09	16.67
	Gm06_48098064_A_C	6	48098064	3.26	6.56	27.23
	Gm07_1246890_T_G	7	1246890	3.50	6.82	6.06
	Gm07_37096617_A_G	7	37096617	3.18	6.45	13.45
	Gm08_8577294_A_G	8	8577294	3.16	6.74	37.26
	Gm08_41844881_T_C	8	41844881	3.16	6.63	22.79
	Gm09_39822766_C_T	9	39822766	3.44	6.68	7.76
	Gm09_45586982_T_C	9	45586982	3.31	6.52	17.03
	Gm10_5026251_T_C	10	5026251	3.03	6.41	37.38
	Gm11_3323629_G_A	11	3323629	3.32	6.96	36.74
	Gm11_8633864_T_C	11	8633864	3.11	6.15	6.11
	Gm12_4025840_T_G	12	4025840	3.02	6.15	14.41
	Gm12_34776200_C_T	12	34776200	3.53	6.93	13.10
	Gm13_6761450_T_G	13	6761450	3.02	6.10	29.91
	Gm13_11463853_T_C	13	11463853	3.26	6.59	29.60
	Gm13_27132590_C_T	13	27132590	3.11	6.16	9.65
	Gm14_13290049_C_T	14	13290049	3.25	6.73	26.61
	Gm15_6415122_A_G	15	6415122	3.41	6.97	31.36
	Gm15_7217705_A_G	15	7217705	3.59	7.17	6.22
	Gm15_7574118_T_C	15	7574118	4.51	9.01	19.73
	Gm15_7721702_G_A	15	7721702	4.25	8.51	9.42
	Gm15_7864348_G_T	15	7864348	4.61	9.19	11.21
	Gm15_8134735_A_G	15	8134735	4.23	8.33	12.78
	Gm15_8263547_G_T	15	8263547	4.92	9.57	13.60
	Gm15_8412363_G_A	15	8412363	4.85	9.56	12.89
	Gm18_51128392_G_A	18	51128392	3.37	6.86	37.10
	Gm18_51659540_A_G	18	51659540	3.44	6.86	35.40
	Gm18_51867289_C_T	18	51867289	3.27	6.53	35.84
	Gm18_58588820_A_C	18	58588820	3.35	6.86	32.27
	Gm19_37932358_C_T	19	37932358	4.84	10.02	18.69
	Gm19_38121212_G_A	19	38121212	3.29	6.87	23.61
GLM-(PCA)	Gm04_17773168_A_G	4	17773168	3.34	6.65	42.52
	Gm06_11098210_C_T	6	11098210	3.93	7.35	16.67
	Gm09_39822766_C_T	9	39822766	3.46	6.44	7.76
	Gm11_3323629_G_A	11	3323629	3.83	7.67	36.74
	Gm12_27424432_A_G	12	27424432	3.11	6.12	7.21
	Gm13_5211326_T_C	13	5211326	3.33	6.42	12.89
	Gm13_6761450_T_G	13	6761450	3.69	7.11	29.91
	Gm13_29418256_C_T	13	29418256	3.04	6.05	29.63
	Gm15_4973977_T_C	15	4973977	4.35	8.33	17.86
	Gm15_7574118_T_C	15	7574118	5.67	10.85	19.73
	Gm15_7721702_G_A	15	7721702	3.84	7.48	9.42
	Gm15_7864348_G_T	15	7864348	4.13	8.02	11.21
	Gm15_8134735_A_G	15	8134735	3.61	6.90	12.78
	Gm15_8263547_G_T	15	8263547	4.40	8.32	13.60
	Gm15_8412363_G_A	15	8412363	4.31	8.23	12.89
	Gm18_51128392_G_A	18	51128392	3.78	7.37	37.10
	Gm18_51659540_A_G	18	51659540	3.60	6.87	35.40
	Gm18_51772288_T_C	18	51772288	3.18	6.10	34.53
	Gm18_51867289_C_T	18	51867289	3.70	7.04	35.84
	Gm19_37932358_C_T	19	37932358	4.50	8.95	18.69
	Gm19_38121212_G_A	19	38121212	3.39	6.75	23.61
MLM_(PCA+K)	Gm06_11098210_C_T	6	11098210	3.44	7.40	16.67
	Gm15_7574118_T_C	15	7574118	3.79	8.08	19.73
	Gm15_7864348_G_T	15	7864348	3.09	6.53	11.21
	Gm15_8263547_G_T	15	8263547	3.35	6.99	13.60
	Gm15_8412363_G_A	15	8412363	3.25	6.83	12.89
	Gm19_37932358_C_T	19	37932358	3.93	9.13	18.69

Of the 35 significant SNPs found under the SMR model, 8 were located on a 2-Mb region of chromosome 15, indicating a strong likelihood of QTL(s) affecting tolerance index based on biomass reduction under SCN infestation in this region (Fig 2A). These 8 SNPs consisted of Gm15_6415122_A_G (LOD = 3.41, MAF = 31.36%), Gm15_7217705_A_G (LOD = 3.59, MAF =, 6.22%), Gm15_7574118_T_C (LOD = 4.51, MAF = 19.73%), Gm15_7721702_G_A (LOD = 4.25, MAF = 9.42%), Gm15_7864348_G_T (LOD = 4.61, MAF = 11.21%), Gm15_8134735_A_G (LOD = 4.23, MAF = 12.78%), Gm15_8263547_G_T (LOD = 4.92, MAF = 13.60%), and Gm15_8412363_G_A (LOD = 4.85, MAF = 12.89%) (Table 3) (Fig 2A). Chromosome 18 harbored a total 4 significant SNP markers mapped on a 7-Mb genomic region. These SNPs consisted of Gm18_51128392_G_A (LOD = 3.37, MAF = 6.86%), Gm18_51659540_A_G (LOD = 3.44, MAF = 6.86%), Gm18_51867289_C_T (LOD = 3.27, MAF = 6.53%), and Gm18_58588820_A_C (LOD = 3.35, MAF = 6.86%) (Table 3) (Fig 2A). These results indicated that the 7-Mb region of chromosome 18 harboring the aforementioned SNPs had a strong likelihood of loci affecting tolerance index based on biomass reduction under SCN infestation in soybean.

#### Generalized linear model (GLM_PCA)

The GLM_PCA model incorporated the principal component (PCA) covariate in its equation. In this study, this model provided a total of 21 significant SNPs (LOD>3) associated with tolerance index based on biomass reduction (Table 3) (Fig 2B). The top 5 SNPs suggested by the GLM_PCA model were Gm15_7574118_T_C (LOD = 5.67, MAF = 19.73%), Gm19_37932358_C_T (LOD = 4.50. MAF = 18.69%), Gm15_8263547_G_T (LOD = 4.40, MAF = 13.60%), Gm15_4973977_T_C (LOD = 4.35, MAF = 17.86%), and Gm15_8412363_G_A (LOD = 4.31, MAF = 12.89%) (Table 3). The R-square values associated with these SNPs ranged from 8.02% to 10.85% with SNP Gm15_7574118_T_C having the highest R-square value.

A total of 7 significant SNPs were mapped on a 3.5-Mb region of chromosome 15 (Table 3) (Fig 2B). These SNPs consisted of Gm15_4973977_T_C (LOD = 4.35, MAF = 8.33%), Gm15_7574118_T_C (LOD = 5.67, MAF = 10.85%), Gm15_7721702_G_A (LOD = 3.84, MAF = 7.48%), Gm15_7864348_G_T (LOD = 4.13, MAF = 8.02%), Gm15_8134735_A_G (LOD = 3.61, MAF = 6.90%), Gm15_8263547_G_T (LOD = 4.40, MAF = 8.32%), and Gm15_8412363_G_A (LOD = 4.31, MAF = 8.23%) (Table 3). A significant portion of the 3.5-Mb region of chromosome 15 containing these significant SNPs overlapped with the 2-Mb region of chromosome 15 that were indicated by the SMR model, thus increasing the likelihood of significant loci controlling tolerance index based on biomass reduction under SCN infestation in this genomic region. In addition, chromosome 18 contained a cluster of 4 significant SNPs mapped on a 740-Kb genomic region (Table 3) (Fig 2B). These SNPs consisted of Gm18_51128392_G_A (LOD = 3.78, MAF = 37.10%), Gm18_51659540_A_G (LOD = 3.60, MAF = 35.40%), Gm18_51772288_T_C (LOD = 3.18, MAF = 34.53%), and Gm18_51867289_C_T (LOD = 3.70, MAF = 35.84%) (Table 3). This region of chromosome 18 also significantly overlapped with that of found with the SMR model.

#### Mixed linear model (PCA + K)

A mixed linear model involving the covariates principal component (PCA) and Kinship (K) was also conducted in order to identify SNP markers associated with tolerance index based on reduction in biomass under SCN infestation in soybean. As expected, fewer SNPs had an LOD greater than 3 compared to the results obtained from the SMR and GLM_PCA models. These SNPs were Gm06_11098210_C_T (LOD = 3.44, MAF = 16.67%), Gm15_7574118_T_C (LOD = 3.79, MAF = 19.73%), Gm15_7864348_G_T (LOD = 3.09, MAF = 11.21%), Gm15_8263547_G_T (LOD = 3.35, MAF = 13.60%), Gm15_8412363_G_A (LOD = 3.25, MAF = 12.89%), and Gm19_37932358_C_T (LOD = 3.93, MAF = 18.69%) with R-square values of 7.40%, 8.08%, 6.53%, 6.99%, 6.83%, and 9.13%, respectively (Table 3) (Fig 2C). Chromosome 15 harbored a total of 4 significant SNPs out of these 6 SNPs. The 4 SNPs were located on an 840-Kb region of chromosome 15, which overlapped with the significant loci indicated by the SMR and GLM_PCA models (Fig 2C). Since this region on chromosome 15 was suggested by all 3 statistical models, the likelihood of having QTL(s) affecting is high. Interestingly, no SNPs having an LOD greater than 3 were found on chromosome 18, unlike the SMR and the GLM_PCA models. If the threshold was 2.60, one SNP marker located on chromosome 18 would be significant.

### Overlapping significant SNP markers between models and candidate genes

The linear regression models upon which the SMR, GLM_PCA, and GLM_PCA_K models were built had different covariates. Despite this discrepancy between models, the results indicated three consistent genomic regions significantly associated with tolerance index based on biomass reduction under SCN infestation in soybean (Fig 2). The SNPs Gm06_11098210_C_T (LOD_SMR = 4.66, LOD_GLM_PCA = 3.93, LOD_GLM_PCA_K = 3.44), Gm15_7574118_T_C (LOD_SMR = 4.51, LOD_GLM_PCA = 5.67, LOD_GLM_PCA_K = 3.79), Gm15_7864348_G_T (LOD_SMR = 4.61, LOD_GLM_PCA = 4.13, LOD_GLM_PCA_K = 3.09), Gm15_8263547_G_T (LOD_SMR = 4.92, LOD_GLM_PCA = 4.40, LOD_GLM_PCA_K = 3.35), Gm15_8412363_G_A (LOD_SMR = 4.85, LOD_GLM_PCA = 4.31, LOD_GLM_PCA_K = 3.25), Gm19_37932358_C_T (LOD_SMR = 4.84, LOD_GLM_PCA = 4.50, LOD_GLM_PCA_K = 3.93) had an LOD greater than 3 regardless of the model (Table 4). These results suggested that the regions harboring these SNPs, especially the 840-Kb region of chromosome 15, had a strong probability of containing QLT(s) affecting tolerance index based on reduction in biomass under SCN infestation in soybean.

**Table 4:** Overlapping significant SNP markers (LOD>3.00) between the Single Marker Regression (SMR), Generalized Linear Model_PCA (GLM_(PCA)), and Mixed Liner Model_PCA_K(MLM_(PCA+K)) models.

**SNP_marker**	**Chromosome**	**Position_(bp)**	**LOD(-log**_**10**_**(p-value))**			**Minor_allele_frequency(%)**
			**SMR**	**GLM_(PCA)**	**MLM_(PCA+K)**	
Gm06_11098210_C_T	6	11098210	4.66	3.93	3.44	16.67
Gm15_7574118_T_C	15	7574118	4.51	5.67	3.79	19.73
Gm15_7864348_G_T	15	7864348	4.61	4.13	3.09	11.21
Gm15_8263547_G_T	15	8263547	4.92	4.40	3.35	13.60
Gm15_8412363_G_A	15	8412363	4.85	4.31	3.25	12.89
Gm19_37932358_C_T	19	37932358	4.84	4.50	3.93	18.69
**SNP_marker**	**Chromosome**	**Position_(bp)**	**R_square**			**Minor_allele_frequency(%)**
			**SMR**	**GLM_(PCA)**	**MLM_(PCA+K)**	
Gm06_11098210_C_T	6	11098210	9.09	7.35	7.40	16.67
Gm15_7574118_T_C	15	7574118	9.01	10.85	8.08	19.73
Gm15_7864348_G_T	15	7864348	9.19	8.02	6.53	11.21
Gm15_8263547_G_T	15	8263547	9.57	8.32	6.99	13.60
Gm15_8412363_G_A	15	8412363	9.56	8.23	6.83	12.89
Gm19_37932358_C_T	19	37932358	10.02	8.95	9.13	18.69
**SNP_marker**	**Chromosome**	**Position_(bp)**	**Gene_ID**	**Functional annotation**		
Gm06_11098210_C_T	6	11098210	Glyma.06G134900	small heat-shock protein (HSP20) family		
Gm15_7574118_T_C	15	7574118	Glyma.15G097500.1	mago nashi family protein		
Gm15_7864348_G_T	15	7864348	Glyma.15G100900.3	Protein phosphatase 2C family protein		
Gm15_8263547_G_T	15	8263547	Glyma.15G105400	Predicted 3-ketosphinganine reductase		
Gm15_8412363_G_A	15	8412363	Glyma.15G107200	NA		
Gm19_37932358_C_T	19	37932358	Glyma.19G121200.1	protein FAR1-related sequence 3-like isoform X1		

Since the 6 aforementioned SNPs were consistent across all 3 models, candidate genes in their vicinity were investigated. These candidate genes consisted of *Glyma*.*06G134900*, *Glyma*.*15G097500*.*1*, *Glyma*.*15G100900*.*3*, *Glyma*.*15G105400*, *Glyma*.*15G107200*, and *Glyma*.*19G121200*.*1* (Table 4). *Glyma*.*06G134900*, *Glyma*.*15G097500*.*1*, *Glyma*.*15G100900*.*3*, *Glyma*.*15G105400*, and *Glyma*.*19G121200*.*1* encoded for small heat-shock protein (HSP20) family, mago nashi family protein, protein phosphatase 2C family protein, predicted 3-ketosphinganine reductase, and protein FAR1-related sequence 3-like isoform X1, respectively, whereas no functional annotation was found for *Glyma*.*15G107200* (Table 4). *Glyma*.*18g225800*, encoding for a homeobox-leucine zipper protein, was found in the vicinity of the SNP maker located on chromosome 18 under the mixed liner model (PCA + K).

### Selection accuracy and efficiency

Selection accuracy and efficiency for the SNPs overlapping between models were calculated. The average selection accuracy for the selected SNPs was 43.00% and ranged between 40.82% and 48.67% (Table 5). The SNP with the highest selection accuracy was Gm06_11098210_C_T, whereas the one with the lowest accuracy among the selected SNP was Gm19_37932358_C_T. Selection efficiency varied from 25.33% to 32.74%, with an average of 28.12% and a standard deviation of 2.58% (Table 5). The SNP with the highest selection efficiency was Gm06_11098210_C_T, and the one with the lowest selection efficiency was Gm15_7574118_T_C (Table 5).

**Table 5:** Genotypic count for the top 78 soybean accessions with the highest tolerance index under SCN infestation, top 78 soybean accessions having the lowest tolerance index under SCN infestation, and selection accuracy and efficiency for the SNPs associated with tolerance index based on biomass reduction under SCN infestation.

	High_tolerance_index							Low_tolerance_index							
SNP	AA	CC	GG	TT	H^a^	Missing^b^	Total	AA	CC	GG	TT	H	Missing	Total	
Gm06_11098210_C_T	0	55	0	7	14	2	78	0	58	0	16	1	3	78	
Gm15_7574118_T_C	0	38	0	18	17	5	78	0	55	0	15	5	3	78	
Gm15_7864348_G_T	0	0	49	8	14	7	78	0	0	66	8	2	2	78	
Gm15_8263547_G_T	0	0	49	9	17	3	78	0	0	64	10	2	2	78	
Gm15_8412363_G_A	9	0	49	0	14	6	78	8	0	68	0	1	1	78	
Gm19_37932358_C_T	0	40	0	18	12	8	78	0	58	0	8	4	8	78	
SNP	Count_for_the_whole_panel							Selection_ accuracy_(%)^c^				Selection_ efficiency_(%)^d^			
	AA	CC	GG	TT	H	Missing	Total	AA	CC	GG	TT	AA	CC	GG	TT
Gm06_11098210_C_T	0	168	0	38	22	6	234		48.67				32.74		
Gm15_7574118_T_C	0	150	0	44	29	11	234		40.86				25.33		
Gm15_7864348_G_T	0	0	176	25	22	11	234			42.61				27.84	
Gm15_8263547_G_T	0	0	171	31	26	6	234			43.36				28.65	
Gm15_8412363_G_A	29	0	175	0	21	9	234			41.88				28.00	
Gm19_37932358_C_T	0	153	0	40	21	20	234		40.82				26.14		

^a^ Count corresponding to heterozygous SNPs.

^b^ Count corresponding to missing SNP data.

^c^ Selection accuracy = 100*[(Number of genotypes having high tolerance index with the favorable SNP allele)/ (Number of genotypes having high tolerance with the favorable SNP allele + Number of genotypes having low tolerance with the favorable SNP allele)].

^d^ Selection efficiency = 100*[(Number of genotypes having high tolerance index with the favorable SNP allele)/(Total number of genotypes having the favorable SNP allele)].

### Genomic selection

**Fig 3:**
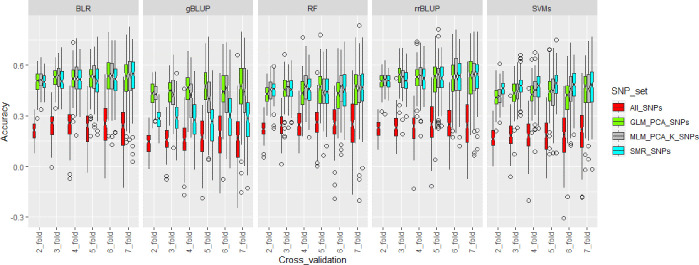
Boxplots showing genomic selection accuracy for SCN tolerance index for biomass reduction under SCN infestation using 5 statistical models: Bayesian Lasso regression (BLR), genomic best linear unbiased predictor (gBLUP), random forest (RF), ridge regression best linear unbiased predictor (rrBLUP), and support vector machines (SVMs). For each model, cross-validation was conducted using different levels (2-fold, 3-fold, 4-fold, 5-fold, 6-fold, and 7-fold) in order to assess the effect of population training size on genomic selection accuracy. At each level of cross-validation, SNP set consisting of all SNPs and SNPs with an LOD greater than 2 based on the GWAS on analysis were used for conducting genomic selection. SMR_SNPs denoted the SNPs from the single marker regression model, GLM_PCA_SNPs represented the SNPs from the generalized linear model, and MLM_PCA_K_SNPs corresponded to the SNPs from the mixed linear model in GWAS. Box plot color coding in the above figure is SNP set-wise. Genomic selection was conducted using a total of 100 replications and empty dots were outliers.

Genomic selection for tolerance index based on biomass reduction under SCN infestation was conducted using statistical models consisting of ridge regression best linear unbiased predictor (rrBLUP), genomic best linear unbiased predictor (gBLUP), Bayesian lasso regression (BLR), random forest (RF), and support vector machines (SVMs) (Table 6). Marker effects were evaluated from all SNPs and the SNPs obtained from GWAS under single marker regression (SMR), generalized linear model (GLM_(PCA)), and mixed linear model (MLM_(PCA+K)) models, respectively. The effect of training population size on genomic selection accuracy was also investigated by conducting cross-validation at different levels with 100 replications for each cross-validation fold. Regardless of the genomic selection model and the size of the training population, the accuracy of genomic selection was higher when the SNPs obtained from GWAS were used (Table 6) (Fig 3). Interestingly, the genomic selection accuracy using the significant SNPs from SMR was not as high as the one obtained from the other GWAS models such as GLM (PCA) and MLM (PCA + K) when the gBLUP model was used for conducting genomic selection (Fig 3). There was not a significant variation in genomic selection accuracy between the SNP set consisting of SMR_SNPs, GLM_PCA_SNPs, and MLM_PCA_K_SNPs for the genomic selection models involving BLR, RF, rrBLUP, and SVMs (Fig 3). Overall, genomic selection accuracy slightly increased when the training population was bigger and plateaued out at a 6-fold cross-validation (Table 6) (Fig 3), corresponding to a training population size of 195 individuals.

**Table 6:** Genomic selection accuracy of tolerance index based on biomass reduction under SCN infestation using 5 statistical models (rrBLUP: ridge regression best linear unbiased predictor, gBLUP: genomic best linear unbiased predictor, BLR: Bayesian Lasso regression, RF: random forest, and SVMs: support vector machines), four SNP sets (all SNPs, SMR_SNPs, MLM_PCA_SNPs, and MLM_PCA_K_SNPs), and different levels of cross-validation (2-fold, 3-fold, 4-fold, 5-fold, 6-fold, and 7-fold) with a total of 100 replications each.

Statistical_models	SNP_set	Summary_statistics	Cross_validation					
			2_fold	3_fold	4_fold	5_fold	6_fold	7_fold
rrBLUP	AllSNPs	Mean	0.22	0.24	0.23	0.26	0.25	0.25
		SD	0.06	0.08	0.09	0.13	0.10	0.14
	SMR	Mean	0.50	0.51	0.52	0.52	0.52	0.53
		SD	0.05	0.07	0.08	0.10	0.12	0.13
	GLM_(PCA)	Mean	0.50	0.54	0.53	0.51	0.52	0.52
		SD	0.05	0.07	0.08	0.12	0.12	0.13
	MLM_(PCA+K)	Mean	0.51	0.52	0.52	0.52	0.53	0.53
		SD	0.05	0.08	0.10	0.11	0.13	0.14
gBLUP	AllSNPs	Mean	0.14	0.17	0.16	0.16	0.19	0.18
		SD	0.07	0.08	0.11	0.13	0.15	0.16
	SMR	Mean	0.28	0.29	0.28	0.26	0.30	0.27
		SD	0.06	0.09	0.12	0.13	0.14	0.15
	GLM_(PCA)	Mean	0.43	0.44	0.46	0.48	0.45	0.45
		SD	0.07	0.08	0.09	0.12	0.13	0.17
	MLM_(PCA+K)	Mean	0.43	0.43	0.40	0.40	0.43	0.44
		SD	0.06	0.11	0.12	0.15	0.16	0.16
BLR	AllSNPs	Mean	0.21	0.24	0.25	0.23	0.24	0.24
		SD	0.06	0.09	0.10	0.11	0.13	0.14
	SMR	Mean	0.50	0.51	0.51	0.50	0.51	0.52
		SD	0.05	0.07	0.09	0.11	0.11	0.15
	GLM_(PCA)	Mean	0.50	0.53	0.52	0.52	0.53	0.52
		SD	0.06	0.06	0.08	0.10	0.11	0.13
	MLM_PCA_K	Mean	0.51	0.53	0.52	0.52	0.52	0.53
		SD	0.06	0.07	0.09	0.10	0.12	0.14
SVMs	AllSNPs	Mean	0.17	0.18	0.18	0.18	0.18	0.21
		SD	0.07	0.08	0.11	0.10	0.14	0.14
	SMR	Mean	0.46	0.48	0.47	0.49	0.47	0.46
		SD	0.05	0.07	0.09	0.10	0.11	0.14
	GLM_PCA	Mean	0.41	0.43	0.43	0.44	0.40	0.45
		SD	0.06	0.08	0.07	0.11	0.13	0.13
	MLM_(PCA+K)	Mean	0.42	0.44	0.45	0.44	0.46	0.45
		SD	0.06	0.07	0.09	0.11	0.12	0.13
RF	AllSNPs	Mean	0.23	0.25	0.25	0.26	0.26	0.25
		SD	0.06	0.08	0.09	0.10	0.12	0.15
	SMR	Mean	0.45	0.46	0.45	0.44	0.45	0.47
		SD	0.06	0.08	0.09	0.11	0.13	0.13
	GLM_(PCA)	Mean	0.43	0.45	0.44	0.46	0.43	0.46
		SD	0.06	0.09	0.09	0.11	0.12	0.14
	MLM_(PCA+K)	Mean	0.45	0.46	0.47	0.44	0.46	0.44
		SD	0.07	0.07	0.09	0.11	0.13	0.16

For rrBLUP, genomic selection accuracy increased to almost 2-fold at each level of cross-validation when the GWAS-derived SNPs were incorporated into the genomic selection model. The highest increase was found at 2-fold cross validation where the genomic selection accuracy was 0.22 when all SNPs were used and was equal to 0.50, 0.50, and 0.51 when the SNP set SMR_SNP, GLM_PCA_SNP, and MLM_PCA_K_SNP were used, respectively (Table 6). At 3-fold and 4-fold cross-validation, genomic selection accuracy was the highest when the SNP set GLM_PCA was used (Table 6). Genomic accuracy was equally high for the SNP set SMR_SNP and MLM_PCA_K_SNP at 5-, 6-, and 7-fold cross validation. For gBLUP, using GWAS-derived SNPs increased genomic selection to almost 3-fold expect for the SMR SNP set (Table 6) (Fig 3). Under the gBLUP model, genomic selection accuracy was the highest when the GLM_PCA SNP set was used. Genomic selection was 0.43, 0.44, 0.46, 0.48, 0.45, and 0.45 at 2-, 3-, 4-, 5-, 6-, and 7-fold cross-validation, respectively, for the GLM_PCA SNP set (Table 6), whereas the accuracy was 0.14, 0.17, 0.16, 0.16, 0.19, 0.18 at 2-, 3-, 4-, 5-, 6-, and 7-fold cross-validation, respectively, when all SNPs were used to perform genomic selection (Table 6). Genomic selection accuracy performed better under the BLR model than the gBLUP model. Genomic selection accuracy was more than 0.50 when the GWAS-derived SNPs were used in the BLR model (Table 6). Unlike the results suggested by gBLUP, genomic selection under the BLR model showed a higher and a more stable result if the SNPs were derived from a GWAS analysis (Fig 3). The SVMs model resulted in a lower genomic selection accuracy than BLR and rrBLUP, but was comparable to gBLUP except for the SNPs derived the SMR model in GWAS (Fig 3). In addition, the genomic selection based on the SVMs model was special in a way that the accuracy was the best when the SNPs from the SMR GWAS model were used, which was not the case for the other genomic selection models (Table 6) (Fig 3). Among the 5 genomic selection models used for predicting tolerance index based on biomass reduction under SCN infestation, the RF model displayed the best accuracy when all SNPs were used (Table 6). However, the selection accuracy under the RF model was lower than rrBLUP and BLR when the GWAS-derived SNPs were used (Table 6) (Fig 3). These results suggested that genomic selection for tolerance index based on biomass reduction under SCN infestation was model-, SNP set-, and training population size-dependent.

## Discussion

A large variation in tolerance index based on biomass reduction due to SCN infection was found among the soybean panel in this study. Biomass of the genotypes M97251029, ALTONA, MN1804CN, M97305077, M97304052, M97205096, M98332108, MN1806SP, and ALPHA was not affected by SCN infection, indicating that these genotypes were tolerant to SCN infection. Among the 9 lines, MN1804CN and ALPHA (PI 564524) contained resistance from PI 88788 and were resistant to HG Type 0 (race 3) [14]; the SCN tolerance in these 2 lines can be due to or partially due to the SCN resistance trait. All other 7 lines were susceptible to race 3 and the SCN population used in this study; the tolerance in these 7 lines must be due to some other traits rather than the traits resistant to SCN development and reproduction. The results suggest that the SCN tolerance traits based on biomass phenotyping is useful in diversifying soybean cultivars with SCN tolerance traits (minimizing SCN damage) in additional to SCN resistance traits (suppressing SCN reproduction).

Genome wide association study (GWAS) has been a powerful tool to identify SCN-resistance loci in soybean [14–16, 36]. A total of 3,782 high quality SNPs were used to conduct GWAS for tolerance index based on biomass reduction under SCN infestation in this study. A total of 35, 21, and 6 SNPs were identified to be associated with tolerance index based on biomass reduction under SCN infestation using the models SMR, GLM (PCA), and MLM (PCA+K). Of which, 6 SNPs overlapped between the 3 models. The discrepancy in terms of the number of significant SNPs found from each model was attributed to the different covariates used upon which each GWAS model was built. PCA accounted for population stratification within the soybean panel investigated in this study. The SMR model does not account for population structure, GLM (PCA) has the ability to reduce false discovery due to population stratification, and MLM (PCA +K) further decreases false discovery rate by incorporation the genetic relatedness between soybean lines, which was denoted as Kinship (K). Both SMR and GLM (PCA) models identified a cluster of significant SNPs found on chromosome 18, which harbored the resistant locus *rhg1* [9]. A few lines involved in the soybean panel used in this study were derived from PI 88788 [14], which has the *rhg1* locus resistant to SCN. The SCN resistant traits can contribute to soybean tolerance under the SCN infection, but the *rgh1* genes have little resistance to the SCN population of HG Type 1.2.3.5.6.7 (race 4) used in this study. Interestingly, the highest GWAS signals were located on an 840-Kb region of chromosome 15. Of the 6 SNPs overlapping between the 3 models, 4 were mapped on chromosome 15. To the best of our knowledge, to date, no SCN-resistant loci have been reported in the vicinity of these SNPs. There were only 16 lines potentially containing SCN resistance of *rhg1* on chromosome 18 from PI 88788. Since the FI of the SCN population on PI 88788 was 28.2 and a soybean derived from PI 88788 generally have higher FI than the source, probably none of these 16 soybean lines could have FI less than 30 and be classified as a soybean resistant to the SCN population used in this study. Therefore, this QTL on this chromosome 15 is probably a sole tolerance trait that promotes soybean growth to minimize biomass reduction by SCN infection, but is unable to suppress SCN reproduction.

One of the most surprising findings reported in this study was the involvement of small heat-shock protein (HSP20) for SCN tolerance based on biomass reduction. In addition to conferring resilience to abiotic stresses such as drought and hot temperatures, small-heat shock proteins (HSP20) have been also suggested to help with plant defense mechanism to pathogen infection [37]. These proteins act as molecular chaperones by assisting with protein folding and other post-translational modifications during pathogen invasion. Park and Seo [37] showed that HSP20 is highly involved in controlling R proteins during plant pathogen attack. Therefore, there could be a link between HSP20 and its involvement in limiting damage of SCN infection in soybean. However, this finding requires additional validation. Our results also indicated a mago nashi family protein to be a good candidate for SCN tolerance. Mago nashi protein is a key component of the exon junction complex (EJC) [38]. The involvement of mago nashi protein in plant defense pathways against pathogens remains poorly understood. However, Gong et al. [38] reported that one of the genes found in the EJC complex is involved in plant growth and development. In this study, we reported loci affecting tolerance index based on biomass reduction under SCN infestation in soybean. We could suggest that the EJC complex might promote plant growth under SCN infestation, resulting in a greater shoot biomass. A phosphatase 2C family protein has been also found to be associated with SCN tolerance in this study. This protein belongs to the class of enzymes requiring Mg^2+^ and Mn^2+^ to be functional. These proteins are highly involved in plant signaling pathways during pathogen infection [39]. One of the pathways involving these proteins that are relevant to biotic stress is the signaling of the hormone abscisic acid (ABA) regulation. Fuchs et al. [39] described that the regulation of ABA upon plant biotic stress contributes to maintaining vegetative growth and modulating plant transpiration. This could explain the fact that some soybean lines used in this study were able to grow and develop under SCN infestation. A predicted 3-ketosphinganine reductase has also been identified as a potential candidate gene for tolerance to SCN infection in this study. This protein has been shown to play key roles in plant response to biotic stress [40]. This protein is an essential element of cell wall and acts as a hormone signaling molecule [40]. Wang et al. [41] demonstrated that the gene encoding for 3-ketosphinganine reductase is upregulated during powdery mildew infection in *Arabidopsis thaliana*. This molecule is significantly involved in the salicylic-acid pathway [41]. Even though no reports have described the direct involvement of 3-ketosphinganine reductase in SCN defense mechanism, we could still speculate that its mechanism in soybean might be similar to the one described during the powdery mildew infection in *Arabidopsis thaliana*. An annotated gene, *Glyma*.*19G121200*.*1*, has also been found in the vicinity of the significant SNPs associated with tolerance index based on biomass reduction under SCN infestation. This gene encodes for FAR1-related sequence 3-like isoform X1. However, to date, there is no report describing the role of this protein in plant defense mechanism against pathogens. Therefore, further analyses are required to elucidate the unknown functions of the proteins transcribed by the annotated genes found in the vicinity of the SNP markers. In addition, further studies are needed for validating the candidate genes prior to their deployment into a marker-assisted selection aiming at improving SCN tolerance in soybean.

Genomic selection has become more and more popular in modern plant breeding [42, 43]. Genomic selection has been proven to be successful in improving genetic gain per unit of time in other studies [35, 44–47]. However, few reports have focused on the potential establishment of genomic selection to unravel the genetic architecture of SCN tolerance in soybean. In this investigation, we performed genomic selection based on 5 statistical models (BLR, gBLUP, rrBLUP, RF, and SVMs) and 4 SNP sets (all SNPs, SMR_SNPs, GLM_PCA_SNPs, and MLM_PCA_K_SNPs). In addition, we have also investigated the effect of training population size on genomic selection accuracy by conducting genomic selection at different levels of cross-validation. Results indicated that genomic selection accuracy was model, SNP set-, and training population size-dependent. This implies that model selection criteria, type of SNPs, population training size are critical components of interest when conducting a genomic selection study. Using GWAS-derived SNPs enhanced the accuracy to almost two-fold. A study conducted by Bao et al. [14] on both association mapping and genomic selection on a total of 282 soybean genotypes for SCN resistance indicated that the use of significant SNPs for conducting genomic selection significantly improved the accuracy of prediction, which was in agreement with the data reported in this current investigation.

In this study we conducted a genome-wide association study (GWAS) to identify SNP markers, and to perform a genomic selection (GS) study for soybean tolerance to SCN infection based on biomass reduction. GWAS has been shown to be successful in studies investigating the genetics of SCN in soybean [48]. Li et al. [49] mapped a 6-Mb region of chromosome 15 to be associated with SCN. In this report, this region has been narrowed to a less than 2-Mb region. In a previous report, we presented the genomic study of soybean tolerance to the SCN infection based on chlorophyll content. Soybean tolerance to SCN may be best characterized by soybean yield response to the SCN infection [48]. However, due to a large number of soybean lines that are needed for the GWAS, it is difficult to conduct a field scale experiment to phenotype the soybean yield response of all lines to SCN in the same field under similar environmental conditions including the SCN infestation level. The greenhouse experiment is the first feasible step to conduct genomic study of SCN tolerance. Biomass in the greenhouse can probably be used to predict yield potential in field, but cautiousness must be taken to extrapolate the greenhouse results to the field setting. We realize that there were some limitations in this greenhouse study. For example, we were unable to conduct the experiment to include all lines at the same time, so it is possible that there were unknown extraneous environmental factors that interfered with our data interpretation. Furthermore, the plants within and between pots appeared to be too crowded for the soybean plants to grow for two months in the greenhouse (S2 Fig), and the plant growth of a line might have affected the plant growth of lines in the neighbor pots. We took consideration of the density of plants per pot and variability of biomass measurements among replicates; if number of plants/pot were reduced, it would probably increase the variability among replicates. It appeared that the plant density of 5 plants/pot was an appropriate design for this study. However, if there is sufficient greenhouse space, it would be better to increase the distance between pots and number of replicates. Nevertheless, to our knowledge, this is the first study of tolerance QTLs to SCN in soybean, perhaps first study of tolerance QTLs to any plant-parasitic nematode based on biomass reduction under nematode infestation. Soybean cyst nematode is mainly managed by using SCN-resistant soybean cultivars, which limit SCN reproduction. SCN-tolerant QTLs may include the QTLs resistant to SCN reproduction, but some QTLs can only affect plant growth, not SCN development and reproduction. This study opened a novel approach to diversify genes that promote plant growth and level off the damage caused by SCN infection. With the advances in genome sequencing and genetic analyses, genomic selection of SCN-tolerant traits is a promising approach to breed SCN-tolerant soybean for enhancing SCN management.

## Conclusions

This study reported the variation in tolerance index based on biomass reduction of a total of 234 soybean genotypes. To the best of our knowledge, this is one of the a few reports investigating SCN tolerance in soybean. In addition to confirming previously reported loci, we identified new loci for SCN tolerance using tolerance index based on biomass reduction. Moreover, we have showed that genomic selection accuracy for SCN tolerance depends on various factors such as statistical models, SNP sets, and training population size.

## Supporting information

S1 Table List of soybean genotypes evaluated for resistance/tolerance to SCN, average adjusted tolerance index based on biomass reduction under SCN infestation, and standard deviation. (XLSX)

S1 Fig Experiment design layout for phenotyping SCN tolerance in greenhouse. The genotypes were replicated four times arranged in four blocks. In each block, the pots were arranged in a split-plot manner, where the genotypes were main plots, and the SCN and no-SCN treatments were sub-plots.
(PPTX)

S2 Fig Greenhouse experiment to phenotype tolerance of soybean to the infection of the soybean cyst nematode based on plant biomass. There were 480 16-cm-diam pots for 60 soybean lines each time of the experiment.
(PPTX)

S3 Fig Kinship plot of 234 soybean genotypes. Values within heat map were obtained from the Kinship matrix of GAPIT).
(PPTX)

## Abbreviations

ANOVA: analysis of variance
BLR: Bayesian lasso regression
gBLUP: genomic best linear unbiased predictor
GLM: generalized linear model
GS: genomic selection
GWAS: genome-wide association study
MLM: mixed linear model
RF: random forest
rrBLUP: ridge regression best linear unbiased predictor
SMR: single marker regression
SVMs: support vector machines
