# Supplementary material for: Genome-wide association study and genomic selection for tolerance of soybean biomass to soybean cyst nematode infestation
Source: PLoS One. 2020 Jul 16;15(7):e0235089. doi: 10.1371/journal.pone.0235089 (PMC7365597; doi:10.1371/journal.pone.0235089)
Supplement: S1 Fig — The genotypes were replicated four times arranged in four blocks. In each block, the pots were arranged in a split-plot manner, where the genotypes were main plots, and the SCN and no-SCN treatments were sub-plots. (PPTX) [file pone.0235089.s002.pptx]

## Slide 1
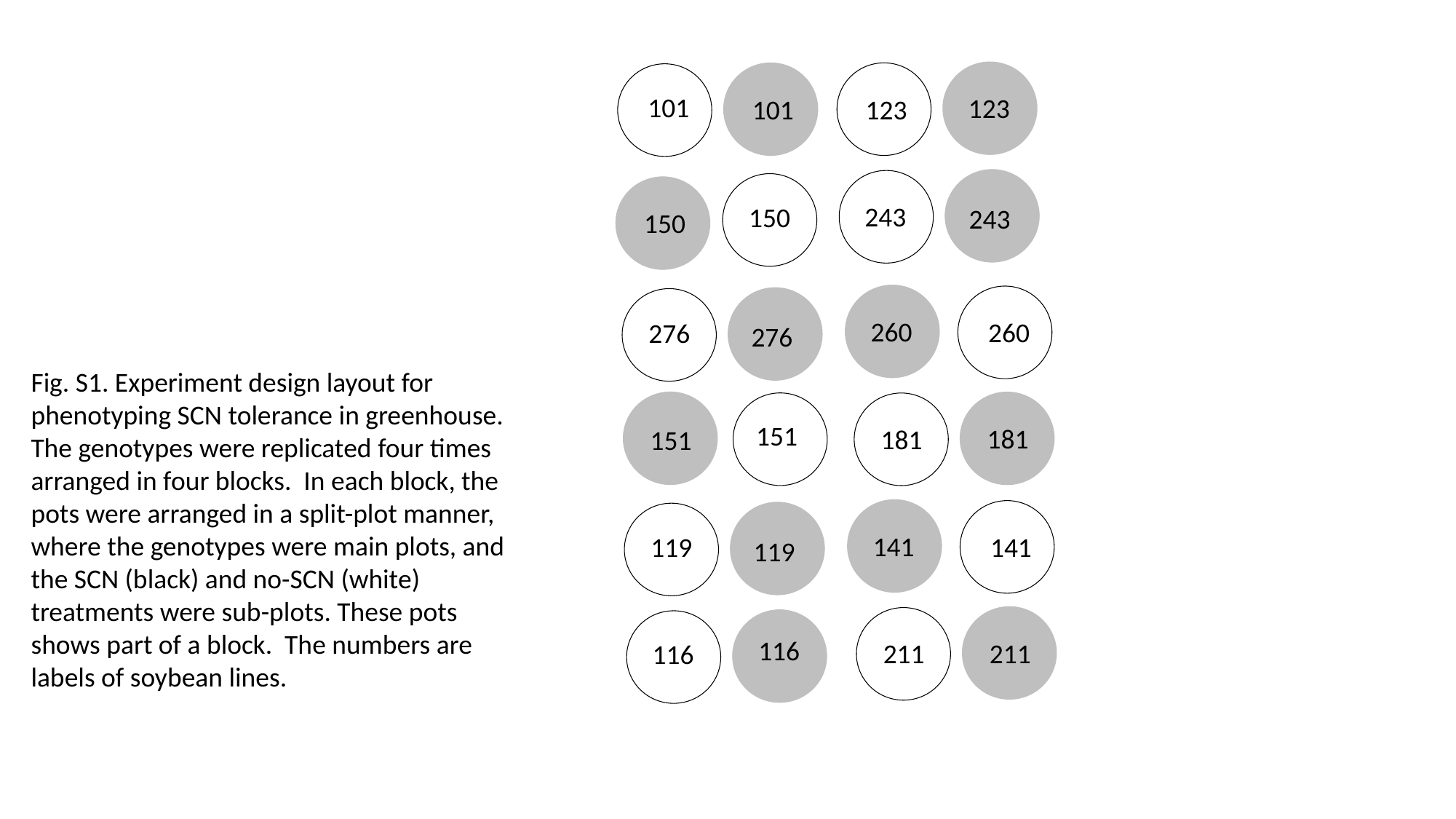

101
123
101
123
243
150
243
150
260
260
276
276
Fig. S1. Experiment design layout for phenotyping SCN tolerance in greenhouse. The genotypes were replicated four times arranged in four blocks. In each block, the pots were arranged in a split-plot manner, where the genotypes were main plots, and the SCN (black) and no-SCN (white) treatments were sub-plots. These pots shows part of a block. The numbers are labels of soybean lines.
151
181
181
151
141
141
119
119
116
211
211
116
