# Supplementary material for: Genome-wide association study and genomic selection for tolerance of soybean biomass to soybean cyst nematode infestation
Source: PLoS One. 2020 Jul 16;15(7):e0235089. doi: 10.1371/journal.pone.0235089 (PMC7365597; doi:10.1371/journal.pone.0235089)
Supplement: S2 Fig — There were 480 16-cm-diam pots for 60 soybean lines each time of the experiment. (PPTX) [file pone.0235089.s003.pptx]

## Slide 1
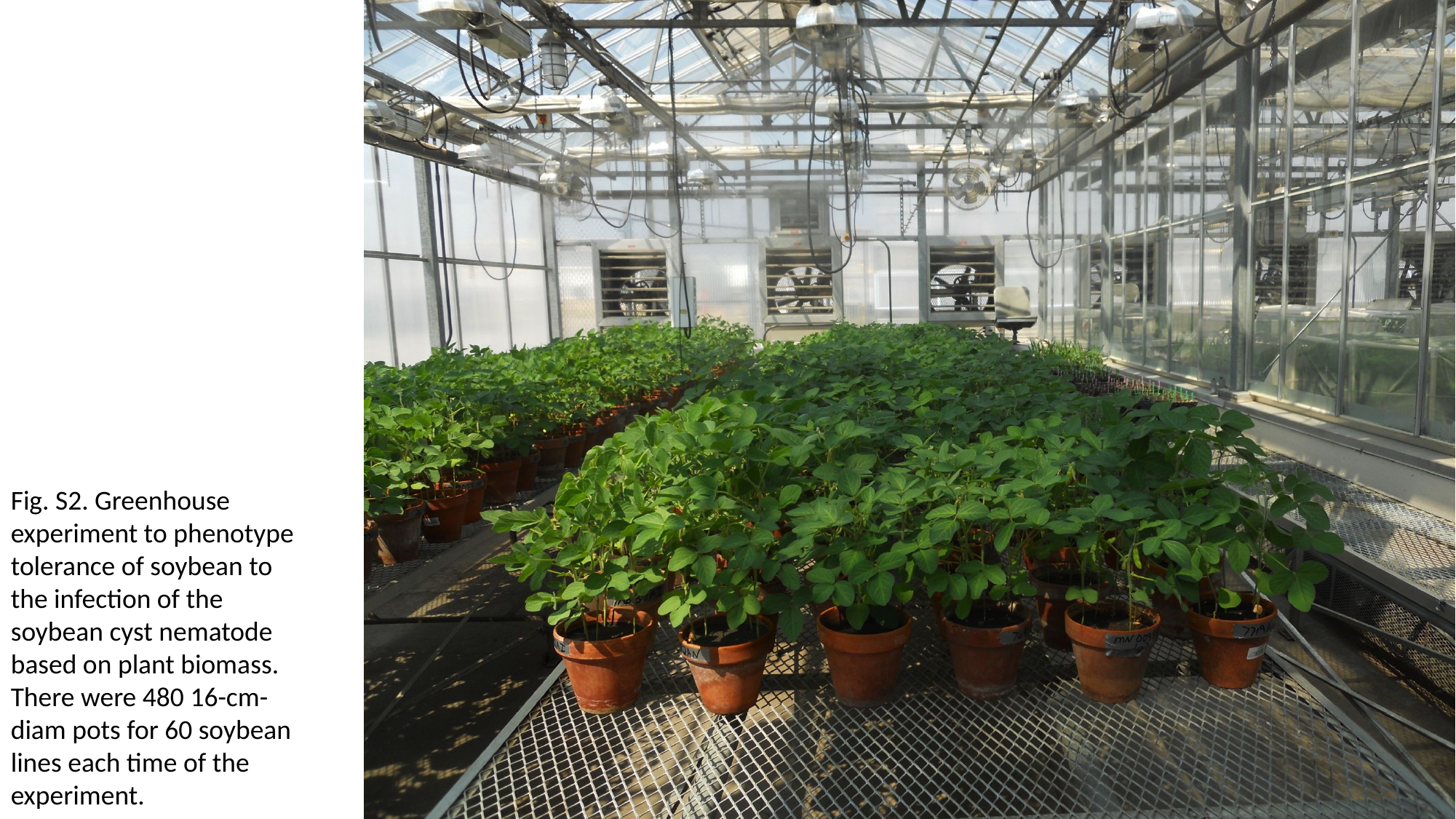

Fig. S2. Greenhouse experiment to phenotype tolerance of soybean to the infection of the soybean cyst nematode based on plant biomass. There were 480 16-cm-diam pots for 60 soybean lines each time of the experiment.
