# Supplementary figures and images for: Genome-wide association study and genomic selection for tolerance of soybean biomass to soybean cyst nematode infestation
Source: PLoS One. 2020 Jul 16;15(7):e0235089. doi: 10.1371/journal.pone.0235089 (PMC7365597; doi:10.1371/journal.pone.0235089)

## Slide 1
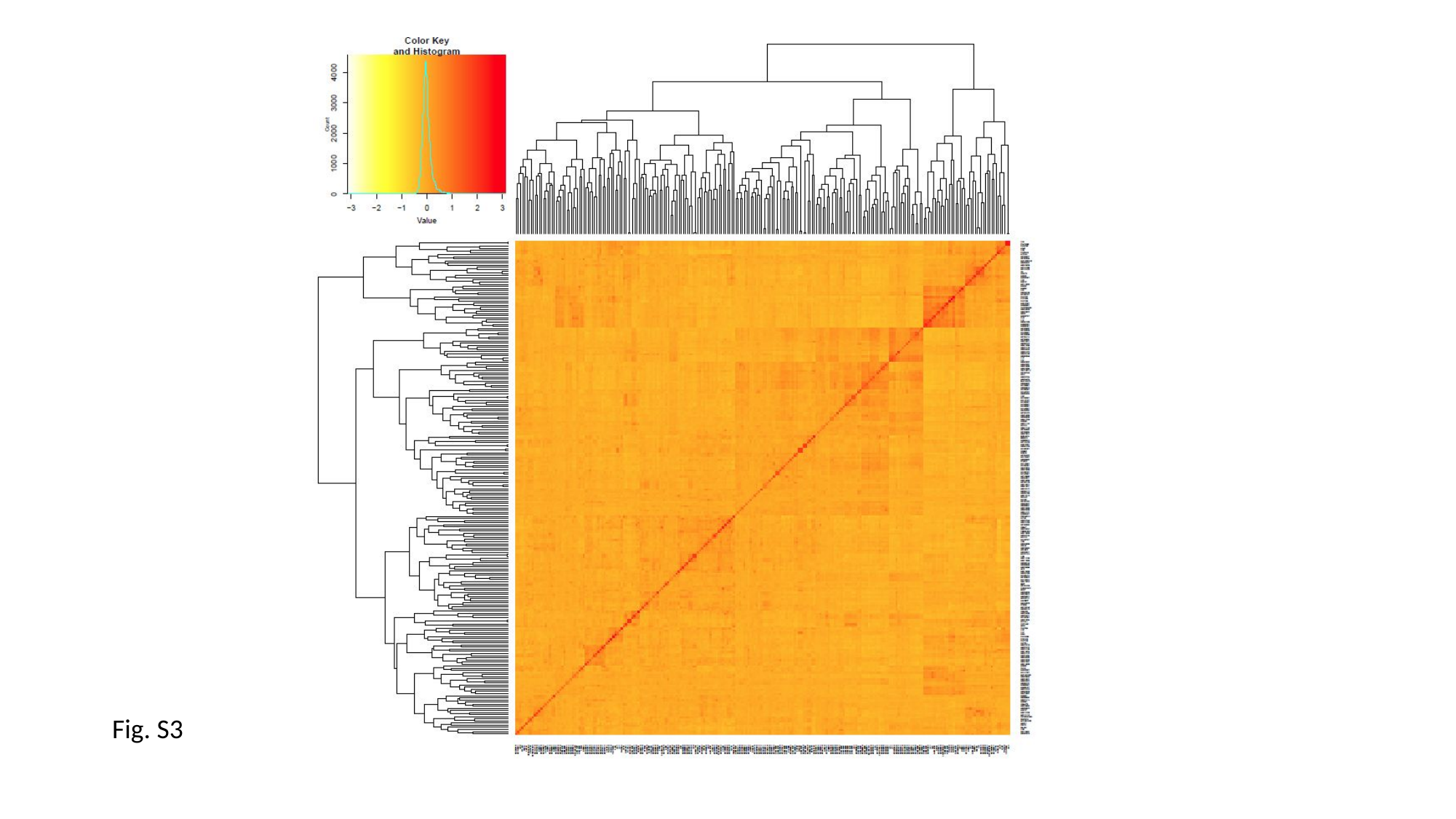

Fig. S3

Supplement: S3 Fig — Values within heat map were obtained from the Kinship matrix of GAPIT). (PPTX) [file pone.0235089.s004.pptx]
